# Predicting the need for calcium modification techniques using computed tomography coronary angiography

**DOI:** 10.1007/s10554-025-03371-4

**Published:** 2025-03-10

**Authors:** David Murphy, Benjamin Hudson, Stephen Lyen, Robert Lowe, Kevin Carson, Sri Raveen Kandan, Daniel McKenzie, Ali Khavandi, Jonathan Carl Luis Rodrigues

**Affiliations:** 1 Cardiology Department, Royal United Hospitals Bath NHS Trust, Combe Park, Bath, Avon, BA1 3NG UK; 2 Department of Health, University of Bath, BA2 7AY Bath, UK; 3 Radiology Department, Royal United Hospitals Bath NHS Trust, Combe Park, Bath, Avon, BA1 3NG UK

**Keywords:** Calcium modification, CT coronary angiography, Calcific coronary artery disease, Chronic coronary syndrome

## Abstract

Calcified coronary arteries pose a challenge to percutaneous coronary intervention (PCI). Calcium modification techniques (CMTs) increase procedural length, complexity and risk. Computed tomography coronary angiography (CTCA) is well suited to calcium identification and quantification and may offer valuable pre-procedural information. We hypothesised that CTCA could predict cases where CMT would be required during PCI. A single centre retrospective review (2021/2022) of consecutive patients who underwent PCI with a preceding CTCA demonstrating a calcified lesion in a major epicardial vessel. Blinded to the PCI strategy CTCA images were re-reviewed and calcium thickness, length, density and circumferential arc quantified. Receiver operating characteristic (ROC) curve for CMT defined optimum cut-off values. Calcium density (> 1000 HU) and calcific arc (> 180°) were proposed as a calcium planning score (CPS_CTCA_), with 1 point assigned per criteria met. 76 PCI procedures were included (72 patients). CMT was used in 53% at the discretion of the operator. Calcific arc, density, length and thickness had an area under the curve (AUC) of 0.74, 0.7, 0.67 and 0.63 respectively. There was a step-wise increase in the proportion of cases requiring CMT with increasing CPS_CTCA_. 0 vs. 1 point; OR 9 (1.1–82, *p* =.04), RR 5 (0.8–36, *p* =.09), 1 vs. 2 points; OR 3.2 (1.1–9.3, *p* =.03), RR 1.6 (1-2.3, *p* =.04), 0 vs. 2 points; OR 30 (3.3–272, *p* =.003), RR of 8 (1.3–54, *p* =.03). The incorporation of CTCA measured calcium density > 1000 HU and calcium arc > 180° into a calcium planning score may help with predicting the need for CMT at the time of PCI.

## Introduction

Computed tomography coronary angiography (CTCA) is now well established in the diagnostic work-up of patients presenting with symptoms suggestive of chronic coronary syndrome (CCS) [1, 2, 3]. Current reporting standards in CTCA deal with the identification of coronary artery disease (CAD), its composition and the impact this has on luminal size, with particular emphasis on CAD providing a substrate for angina [4]. CTCA has the potential to provide much more than diagnostic information alone. More recently its role in pre-procedural planning of percutaneous coronary intervention (PCI) has become increasingly recognised [5]. CTCA can provide information on best angiographic angles to visualise obstructive disease, angulation into the left main stem, vessel tortuosity, side branch significance and risk of loss during PCI. In addition lesion location, length and composition can all be combined to provide a robust pre-procedural assessment of the patient [6]. 

Coronary artery calcification (CAC) is present in approximately 30% of PCI procedures with a prevalence that is increasing [7, 8]. CAC often poses a distinct challenge to PCI and is associated with a poorer prognosis [9]. Intervention on lesions where calcific disease is not recognised or where the degree of calcification is underappreciated can lead to coronary artery perforation. Although a rare (0.6%) complication, it can be fatal [10]. CAC can restrict the passage of stents and balloons across stenoses. In some cases producing uncrossable lesions. Calcific plaque reduces arterial compliance and therefore can prevent optimum stent expansion and strut apposition with the arterial wall [11]. This can lead to higher target vessel failure (TVF), target vessel myocardial infarction (MI) and greater revascularisation rates [12]. 

A number of adjuncts are now available to assist with the preparation of calcific plaque including, rotational atherectomy (RA), orbital atherectomy (OA), non-compliant balloons, cutting balloons, scoring balloons, excimer laser and intravascular lithotripsy [13]. These are used prior to stent insertion. Such preparation allows for optimum stent expansion and is key to a successful interventional procedure. These calcium modification techniques (CMTs) often require advanced operator skills, longer interventional time, are higher risk procedures and may require larger arterial access.

Angiography is a poor imaging modality to identify and/or quantify calcific burden prior to intervention, with fluoroscopy only detecting CAC in 10–35% of cases [14]. Intravascular imaging, such as intravascular ultrasound (IVUS) and optical coherence tomography (OCT) are valuable adjuncts at the time of angiography, providing detailed information as to the extent of the CAC and facilitating measurements such as calcium length and thickness [15]. Additionally, and importantly, published guidance for both IVUS and OCT have incorporated several imaging parameters to produce an overall calcium ‘score’. This score provides guidance to the operator as to whether the use of CMT should form part of the PCI strategy [16, 17, 18]. Both IVUS and OCT add additional time to a procedure and require operator training and experience to be used appropriately. Their use is not widespread, with published UK rates of approximately 15% and 3% for IVUS and OCT respectively [19]. 

The diagnosis of calcific plaque and the likelihood of needing CMT prior to any planned PCI may offer valuable information to the interventional team. CTCA is extremely well suited for the identification and quantification of CAC, additionally many patients will have undergone CTCA prior to any elective angiography providing a unique opportunity to plan PCI appropriately. We hypothesised that CTCA could predict cases that needed calcium modification. We aimed to test this in a cohort of patients who underwent PCI with a preceding CTCA.

## Methods

### Participants

**Fig. 1:**
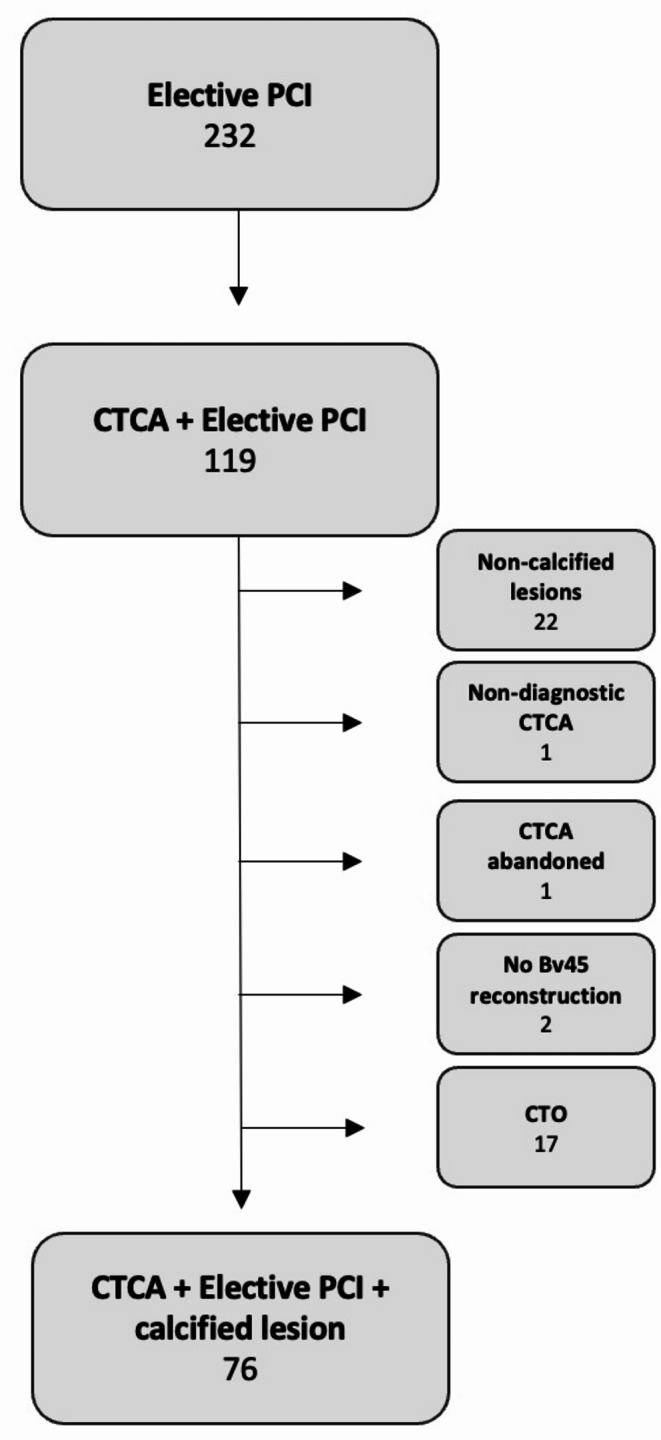
Study flow diagram. CTCA = computed tomography coronary angiogram.PCI = percutaneous coronary intervention.CTO = chronic total occlusion

This study involved a retrospective review of consecutive elective patients that underwent PCI for CCS over a 2 year (2021/2022) period at our institution. This list was cross referenced for any cases which had a preceding CTCA as part of their diagnostic work-up. Electronic records were manually searched to complete demographic details and cardiovascular risk factors (Fig. 1).

### CTCA image acquisition protocol

CTCA were acquired as per normal routine clinical care. Standard departmental protocol includes the administration of intravenous beta blocker as appropriate to target a heart rate of < 60 bpm with sub-lingual nitrate given as the standard vasodilator. CTCA Studies were acquired with a 128-slice CT scanner (Siemens SOMATOM Drive, Siemens Healthineers, Erlangen). Imaging protocol involved a test bolus technique (12 ml Niopam 340 at 6 ml/sec) then full acquisition (60 ml Niopam 340 at 6 ml/sec). A slice thickness of 0.6 mm, a pitch sequential scan feed of 34.5 mm, rotation time 0.28s, and a tube voltage reference of 100kVp with automated kV modulation and 220 reference mAs with automated tube current modulation. Default CTCA reconstructions were: cardiac field of view 0.6 mm coronary vascular reconstruction kernel (Bv38, Siemens Healthineers), 0.6 mm sharp vascular reconstruction kernel (Bv45, Siemens Healthineers) and 0.6 mm raw axial reconstructions without automated smoothing between steps (Truestack I30f, Siemens Healthineers). CTCAs were reported through routine clinical care by a consultant radiologist with at least 5 years’ experience. CTCAs which were non-diagnostic, did not have the appropriate reconstruction kernel or where a chronic total occlusion (CTO) was diagnosed were excluded (see study flow diagram 1).

### PCI

PCI was performed either on an Innova IGS 520 monoplane (GE Healthcare) or an Innova 2121 Biplane (GE Healthcare) with standard acquisition settings of 15 fps, 60 kV and 3.7 mA and fluoroscopy of 7.5 fps, 67 kV and 0.3 mA. Images were acquired according to standard practice with at least 2 projections obtained per vessel distribution. The use of intravascular imaging and the choice of CMT were at the discretion of the operator at the time of angiography. All operators were consultant interventional cardiologists with at least 5 years’ interventional experience.

### CTCA re-review

**Fig. 2:**
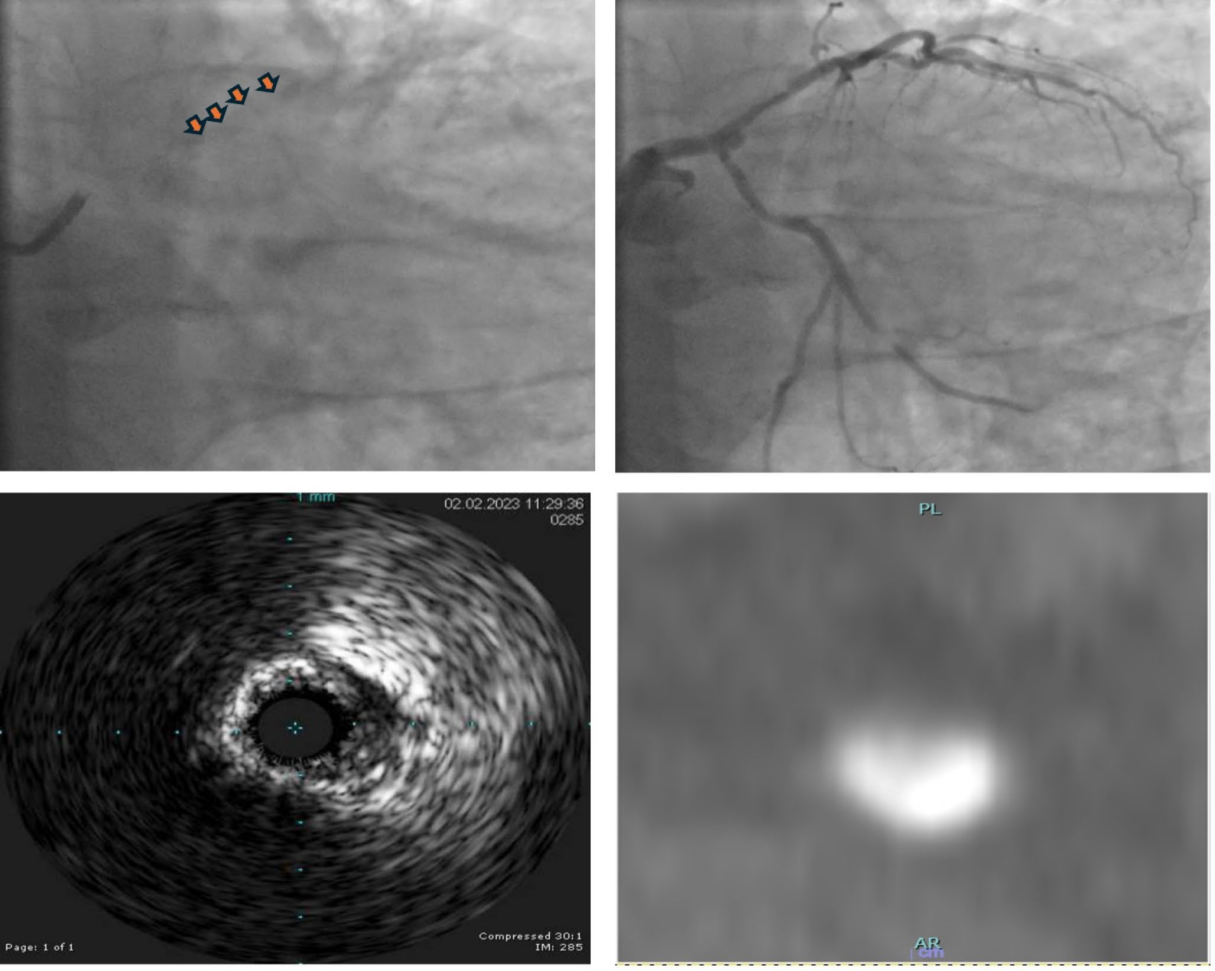
A representative example of a diagnostic angiogram pre-contrast injection with calcified CAD outlined with arrow heads (**a**), and post contrast injection with severe proximal LAD stenosis (**b**). Subsequent IVUS imaging with a 240° arc of coronary artery calcification (**c**). Cross-sectional CTCA imaging from the same location with 240° of calcification and calcium density of 1619 HU (**d**). CAD-coronary artery disease. LAD = left anterior descending.IVUS = intravascular ultrasound imaging. CTCA = computed tomography coronary angiogram.HU = Hounsfield units

Sub-specialist cardiothoracic radiologists with > 10 years’ experience then re-reviewed the CTCA images of the included cases in order to standardise CTCA-related calcium measurements. The experts were blinded to the PCI strategy including the use of CMT but the segment of artery that underwent PCI was disclosed to them. A qualitative assessment of a lesions calcific volume was made and those with > 50% were included. Only the lesions that corresponded to the stented segment were included. The calcific plaque in the region of interest with the highest calcific burden was chosen. Calcium length, thickness, degree of circumferential disease and density were selected for further re-review in this study. These measurements form part of intravascular imaging parameters for assessing the need for CMT. Intravascular imaging utilises a scoring system whereby parameters are assigned one point when present with increasing number of points associated with a greater likelihood that a lesion will require CMT. We presented CTCA parameters in a similar fashion. All measurements were made on dedicated sharp reconstruction kernel using Syngo via (Bv45, Siemens Healthineers) with optimised CT windows. Calcium length and width were measured on longitudinal and cross-sectional reconstructions respectively. The degree of circumferential calcification was subdivided into 4 categories for simplicity purposes - <90°, 90–180°, 180–270° and > 270° on arterial cross-section. A measure of maximal calcium density, in Hounsfield units (HU), was also obtained. A representative example is given in Fig. 2.

### Statistical analysis

The Kolmogorov-Smirnov test was used to assess the data for normality of distribution. Continuous data is presented as mean and standard deviation (SD) or median and interquartile range (IQR) where data distribution was significantly divergent from normality. Categorical data is presented as percentage and frequency. Differences between continuous variables was assessed with Mann-Whitney U testing. Categorical variables were compared using a Fisher exact test. Univariable logistical regression analysis and subsequently multi-variable logistical regression analysis was undertaking using step-wise entry. Receiver operator characteristic (ROC) curve analysis defined the optimum diagnostic ‘cut-off’ values. Such values were defined based on minimising the difference between the positive and negative predictive values. Both odds ratio (OR) and relative risk (RR) were calculated with significance established at *p* <.05.

### Ethics

This study involved a retrospective review of clinically indicated PCI procedures. As per the NHS Research Authority decision tool [20] no written informed consent was obtained and no ethical committee approval was deemed necessary. The study was registered as a service evaluation with the trusts audit department waiving the need for formal written consent. Patients and public were not involved in the design, conduct, reporting or dissemination of our research.

## Results

### Patient and procedural details

Over the study period 232 elective PCI procedures were undertaken. 45% (105/232) had CMT. 33% (76/232) had a preceding CTCA and were included in the study involving 72 patients. CMT was used in 53% (40/76) of these procedures. There were no statistically significant differences in the cardiovascular risk factor profiles of those patients that had CMT and those that did not. The most common vessel to undergo PCI was the proximal LAD in both groups. Overall 61% (46/76) of cases involved intravascular imaging. Cases where CMT was used involved greater use of intravascular imaging (*p* =.03). The most commonly used CMT modalities were cutting balloon, non-compliant balloon and intravascular lithotripsy, used in, 38% (15/40), 35% (14/40) and 28% (11/40) of cases respectively. Patient demographic and procedural details are included in Tables 1 and 2 below.

**Table 1:** Patient demographics

	Total (*n* = 76)	CMT (*n* = 40)	No CMT (*n* = 36)	*p*-value
Mean age, years (SD)	71 (±9)	72 (±9)	70 (±9)	0.3
Male, % (n)	76 (58)	78 (31)	75 (27)	1
HTN, % (n)	53 (40)	53 (21)	53 (19)	1
Diabetes Mellitus, % (n)	20 (15)	18 (7)	22 (8)	0.8
Smoking history, % (n)	45 (34)	43 (17)	47 (17)	0.8
Dyslipidaemia, % (n)	47 (36)	38 (15)	58 (21)	0.1
Family history of premature CAD, % (n)	22 (17)	20 (8)	25 (9)	0.8
Mean BMI, kg/m^2^ (SD)	30 (±5.5)	29.3 (±4.7)	30 (±6.4)	0.7

SD = standard deviation.CAD = coronary artery disease.BMI = Body mass index

**Table 2:** Procedural characteristics

	Total (*n* = 76)	CMT (*n* = 40)	No CMT (*n* = 36)	*p*-value
LMS PCI, % (n)	4 (3)	5 (2)	3 (1)	1
LAD* PCI total, % (n) Prox LAD Mid LAD Distal LAD	72 (55) 67 (37) 33 (18) 0 (0)	73 (29) 69 (20) 31 (9) 0 (0)	72 (26) 75 (12) 25 (4) 0 (0)	1
LCx PCI total, % (n) Proximal LCx Mid LCx Distal LCx	4 (3) 33 (1) 66 (2) 0 (0)	3 (1) 0 (0) 100 (1) 0 (0)	6 (2) 50 (1) 50 (1) 0 (0)	0.6
RCA PCI total, % (n) Prox RCA Mid RCA Distal RCA	20 (15) 33 (5) 53 (8) 13 (2)	20 (8) 38 (3) 62 (5) 0 (0)	19 (7) 29 (2) 42 (3) 29 (2)	1
Mean difference in months between CTCA and PCI (IQR)	6.1 (3-7.3)	5.8 (3.5–7.3)	6.3 (3-7.3)	0.9
Intravascular imaging, % (n)	61 (46)	73 (29)	47 (17)	0.03
CMT modality, % (n)				
Non-compliant balloon (NCB)	-	28 (11)	-	-
Cutting balloon (CB)		28 (11)		
Intravascular lithotripsy (IVL)	-	13 (6)	-	-
Combination therapy	-	15 (6)	-	-
Rotational atherectomy (RA)		10 (4)		
Scoring balloon (ScB)	-	3 (1)	-	-
Excimer laser (ELCA)	-	3 (1)	-	-

LMS = left main stem.PCI = percutaneous coronary intervention.Lcx = left circumflex.RCA = right coronary artery.CTCA = computed tomography coronary angiogram.CMT = calcium modification therapy. *1 PCI to D1 artery included

### Individual CTCA measurements

**Fig. 3:**
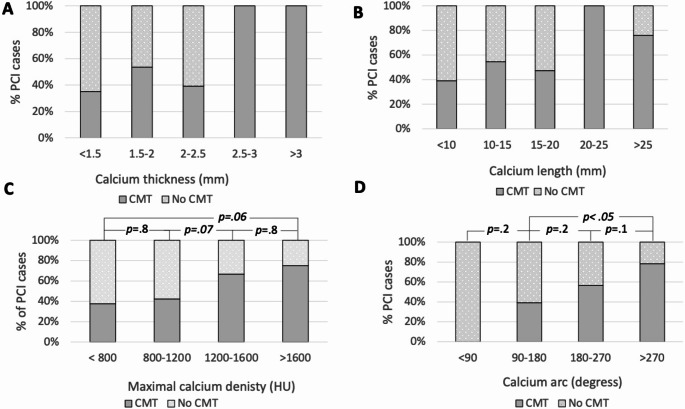
% of PCI cases requiring CMT separated by imaging parameters measured at CTCA. A = calcium thickness measured at CTCA. B = Calcium length measured at CTCA. C = Calcium density measured at CTCA. D = Calcium arc measured at CTCA. CMT = calcium modification therapy.PCI = percutaneous coronary intervention

Between group differences in CTCA parameters is given in Table 3. Calcific lesions that required CMT had a higher maximal calcium density [1287 HU vs. 974 HU (*p* =.003)] and > 270° of calcific arc (*p* =.005). No cases with a calcific arc < 90° required CMT. There was a non-significant step wise increase in the proportion of cases requiring CMT with increasing calcium density and calcific arc angle (Fig. 3). All covariates were significant on univariable logistic regression analysis testing with calcium length, thickness, density and quadrant yielding *p*-values of 0.02, 0.02, 0.001, and < 0.001 respectively. Multivariable logistic regression model was statistically significant, χ2(71) = 23, *p* <.001. Nagelkerke R^2^ of 0.35.

Receiver operating characteristic (ROC) curve demonstrated an area under the curve (AUC) of 0.63 for calcium thickness, 0.67 for calcium length, 0.7 for calcium density and 0.74 for degree of circumferential calcification. An optimum ROC cut-off value for calcium thickness, length, density and calcium arc was found to be 2 mm, 12 mm, 1037 HU and > 180° respectively, with each individual parameters sensitivity, specificity, positive predictive value (PPV) and negative predictive value (NPV) outlined in Table 4.

**Table 3:** Between group differences in CTCA findings

	Total (*n* = 76)	CMT (*n* = 40)	No CMT (*n* = 36)	*p* value
Mean CTCA calcium density, HU (SD)	1109 (335)	1215 (320)	992 (316)	0.003
Mean CTCA calcium thickness, mm (SD)	1.7 (0.6)	1.9 (0.6)	1.7 (0.4)	0.06
Median CTCA calcium length, mm (IQR)	10 (6–14)	11 (7–18)	7.5 (5–12)	0.02
Calcium arc %, (n) < 90° 90–180° 180–270° > 270°	9 (7) 30 (23) 30 (23) 30 (23)	0 (0) 23 (9) 33 (13) 45 (18)	19 (7) 39 (14) 28 (10) 14 (5)	0.03 0.1 0.8 0.005

CMT = calcium modification therapy

**Table 4:** Sensitivity, specificity, positive predictive and negative predictive values of CTCA based parameters for the use of calcium modification therapy at the time of percutaneous coronary intervention

Imaging parameters	Sensitivity	Specificity	Positive predictive value (PPV)	Negative predictive value (NPV)
Calcium thickness > 2 mm	27.5%	88.9%	73%	52.5%
Calcium length > 12 mm	42.5%	77.8%	68%	54.9%
Calcium density > 1000 HU	80%	52.8%	65.3%	70.4%
Calcium arc > 180°	77.5%	58.3%	67.4%	70%

### Calcium planning score (CPSCTCA)

**Fig. 4:**
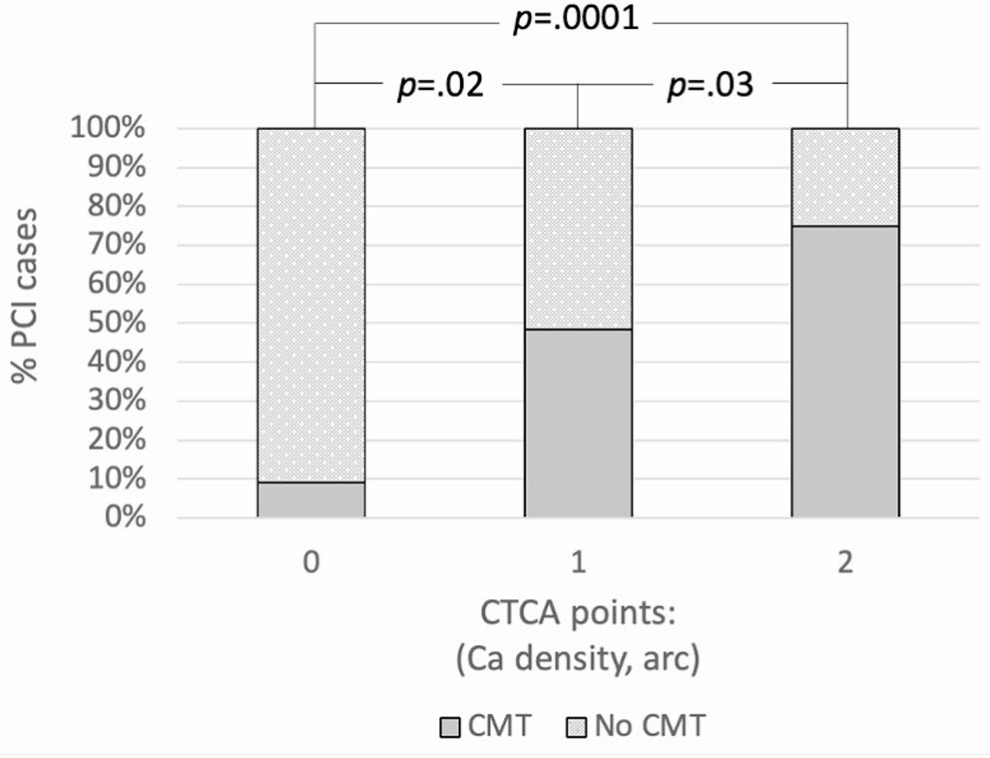
% of PCI cases requiring CMT based on a calcium planning score. incorporating calcium density > 1000 HU and calcific arc > 180° CMT = calcium modification therapy.PCI = percutaneous coronary intervention.CPS_CTCA_=calcium planning score based on CTCA

**Fig. 5:**
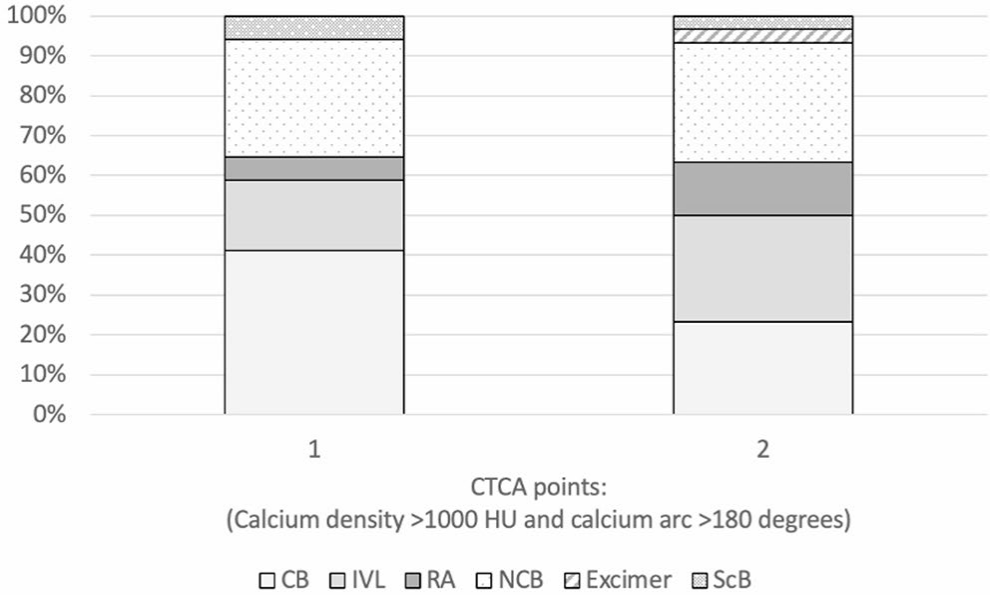
Types of calcium modification therapy techniques at different CTCA CAC score. CB = cutting balloon.IVL = intravascular lithotripsy.RA = rotational atherectomy.NCB = non-compliant balloon.Excimer = Excimer laser.ScB = scoring balloon

Using calcium density > 1000 HU and calcific arc > 180° a CTCA based calcium planning score (CPS_CTCA_) was calculated with 1 point assigned per criteria met. There was a step-wise increase in the proportion of cases requiring CMT with increasing CPS_CTCA_ (Fig. 4). An increase from 0 points to 1 had an OR 9 (1.1–82, *p* =.04) and RR 5 (0.8–36, *p* =.09). An increase from 1 point to 2 had an OR 3.2 (1.1–9.3, *p* =.03) and RR 1.6 (1-2.3, *p* =.04). A CPS_CTCA_ score of 0 versus 2 points had an OR of needing CMT of 30 (3.3–272, *p* =.003) with a RR of 8 (1.3–54, *p* =.03) (Fig. 5).

## Discussion

In this study we looked at CTCA predictors of the need for CMT in patients subsequently listed for PCI. Both calcium density (> 1000 HU) and calcium arc (> 180°) were found to be positively associated with the need for CMT at the time of PCI. A scoring system (CPS_CTCA_) using both parameters demonstrated a step wise increase in the prevalence of CMT use. The additional measurements of calcium length and thickness failed to add to the predictive model. Both parameters are easily measurable at the time of CTCA reporting.

Before the advancements in non-invasive CTCA technology it was common place for a diagnostic invasive coronary angiogram to be undertaken prior to patients being referred for subsequent PCI by a trained interventional cardiologist. PCI was often then routinely planned based on the diagnostic angiogram. CTCA has rapidly evolved and replaced significant proportions of diagnostic angiography but its role in PCI planning has lagged behind. This is in stark contrast to other disciplines such as structural interventional cardiology where it plays a well-established pivotal role [21]. A pre-procedural planning meeting may well offer valuable information for the interventional team prior to planned care. Manipulation of 3D CT modelling of the coronary anatomy can help to highlight coronary tortuosity, bifurcation angles and facilitate accurate lesion length measurements without the foreshortening associated with invasive angiography. Coronary ostia location and angulation can be assessed as can the aortic root potentially facilitating guide catheter choice [5]. An interactive PCI planning tool using baseline CTCA coronary geometry data has recently been developed (HeartFlow Inc., Redwood city, CA). This allows for virtual stent placement and subsequent re-calculation of post PCI fractional flow reserve (FFR) [22]. This has been validated against invasive FFR measurements and clinical experience with its use has recently been published [23] including calcific lesions [24]. Increasing use of artificial intelligence based plaque analysis may allow for the rapid standardisation of calcium burden and integration of the parameters measured in this study [25]. Adequate interventional time, the necessary equipment and appropriately trained personnel could be available to undertake procedures where CTCA is predicting more complex cases, potentially helping to improve the efficiency of the angiography suite.

With the potential for patients to have a CTCA prior to a clinic appointment there is the opportunity to maximise the yield of the patient clinician interaction through bespoke PCI consent given the added complexity to the PCI procedure incurred by the use of CMT [26]. 

The choice of CTCA parameters chosen to measure in this study were based on published intravascular imaging scoring systems [17, 18]. These have taken into account other aspects of the plaque morphology such as depth of calcification and vessel diameters. Such parameters may add further value but CTCA is a poor imaging modality for such features and therefore were omitted from this study. We felt that the parameters chosen represent pragmatic easily measurable indices at the time of CTCA. The use of pre-procedural CPS_CTCA_ reporting should not be seen as a replacement for intravascular imaging but a complimentary tool. A high CPS_CTCA_ may prompt the operator to undertake imaging where under other circumstances they may not have done. This study has grouped all CMTs together. Although all provide a means of dilating the target lesion, rotational atherectomy, orbital atherectomy and excimer laser are sometimes chosen in cases where the lesion itself prevents the passage of balloons and/or imaging probes [27]. This may relate more to the plaque geometry than its morphology.

This was a single centre study with limited numbers of procedures and a large proportion of the CMTs used would be considered less aggressive. This work did not however set out to establish which method of CMT should be used but to identify cases where there was a high likelihood of their requirement, thereby pre-arming the interventional team. The choice to undertake intravascular imaging and any PCI strategy was at the discretion of the operator. We excluded CTO procedures from our study but the important role of CTCA in planning such procedures is well established and can provide valuable information for the operator [28]. A standardised post procedural imaging to measure minimal stent area (MSA) to assess the adequacy of stent expansion has not been undertaken therefore PCI without CMT may well have resulted in sub-optimal results. Further work in a prospective fashion with mandatory intravascular imaging would be useful to provide more insights into the role CTCA calcium analysis could have for pre-procedural PCI planning. This could extend not only to the prediction of CMT use but the type of CMT most likely to succeed.

## Conclusions

A grading of the degree of circumferential calcific plaque and calcium density of coronary artery lesions are important features in determining the likelihood of PCI procedures requiring calcium modification techniques. Consideration should be given to their inclusion in CTCA reports where PCI is likely in order to provide additional important information to the interventional team.

## Data Availability

No datasets were generated or analysed during the current study.
